# Enhancing Mindfulness and Well-Being in Higher Education

**DOI:** 10.1007/s42413-021-00118-6

**Published:** 2021-05-18

**Authors:** Randy K. Barker, Lori P. Tuominen, Mimi Rappley Larson, Mary E. Lee-Nichols, Gloria Eslinger, Kristine L. Patterson, Shevaun L. Stocker

**Affiliations:** 1 Pruitt Center for Mindfulness and Well-Being, University of Wisconsin-Superior, Superior, WI USA; 2 Human Behavior, Justice and Diversity Department, University of Wisconsin-Superior, Superior, WI USA; 3 Department of Education, University of Wisconsin-Superior, Superior, WI USA; 4 Visual Arts Department, University of Wisconsin-Superior, Superior, WI USA; 5 Department of Campus Recreation, University of Wisconsin-Superior, Superior, WI USA

**Keywords:** Mindfulness, Well-being, Higher education, Well-being model, Pruitt center

## Abstract

This article outlines the steps taken to establish the University of Wisconsin-Superior’s Pruitt Center for Mindfulness and Well-Being. Major historical components include: gaining momentum; securing funding; developing mission and vision statements; launching the Pruitt Center; and recounting the services, programs, and impacts achieved to date. Through outlining experiences and lessons learned, others in higher education looking to enhance the well-being of their campus communities could benefit, regardless of whether creating a center is their goal. The process and rationale for creating and adopting the PERMANENT Model of Well-Being is also provided. Comparisons are made regarding the similarities and differences between the PERMANENT Model and two existing models: the PERMA Model and the Universidad Tecmilenio Well-being in Happiness Ecosystem. Also depicted is the intention concerning: 1) describing each domain of the PERMANENT Model of Well-Being, including Present Moment Awareness, the model’s foundation; 2) the meaning behind the PERMANENT acronym, inspiring the notion of long-lasting well-being; 3) including the greater community; and 4) the model’s process of *learn, experience, reflect,* and *repeat*, a reminder that all learning takes effort and practice. This process is supported by current mindfulness and well-being research, specifically as it relates to higher education.

The purpose of this article is two-fold: first, to describe the deliberate steps taken at the University of Wisconsin-Superior (UW-Superior) toward creating a mindfulness and well-being culture that would permeate all aspects of campus life and into the surrounding community. Creating a center within the university, one that would provide services and facilitate programs, became essential to accomplishing the long-range goals of well-being. The second purpose of this article is to provide details about how—and why—it was necessary to create a unique well-being model rather than using an existing one to meet the needs and demands specific to UW-Superior, and ultimately serving as the foundation for the Pruitt Center for Mindfulness and Well-Being (also known as the Pruitt Center) (Pruitt Center for Mindfulness & Well-Being, n.d.). Examples of the most impactful services offered will also be included.

## The History of the Pruitt Center for Mindfulness and Well-Being

This article provides the history of the work surrounding well-being on the campus prior to and including the establishment of the Pruitt Center. The intent is that this experience, including a description of the step-by-step process undertaken, will be beneficial for others in higher education who may also be interested in creating similar services and/or a center.

### Identifying Champions and Increasing Knowledge

In 2015, a small group of faculty and staff began informal discussions with others on campus who shared a passion and intense interest in mindfulness and well-being, in both their personal and professional lives. The desire was to include those who could have the greatest impact across our campus community. A deliberate effort was made to identify representatives from all levels – faculty, staff, student affairs professionals, and upper administration officials, including the Provost. The involvement of the Provost was vital in gaining campus momentum and support. These campus representatives became the champions.

Once champions had been identified, the goal was to provide them with knowledge of best practices and training from experienced academic researchers and other science-based experts. For the UW-Superior campus, this was accelerated by sending the four identified champions (the Provost, two faculty members, and one student affairs professional) to the University of California Berkeley Greater Good Science Center (GGSC) Summer Institute for Educators in Social and Emotional Learning in July of 2015. Funding for this critical phase was provided by a UW-Superior alumna and her husband, whose relationship with the university and a commitment to well-being supported this endeavor. The shared GGSC experience was the catalyst for developing a foundational philosophy to communicate to the campus. Concurrently, the university was involved in a turbulent transition of program and budget cuts, along with declining morale. The belief was that implementing innovative ways to increase the well-being of everyone on campus would help to improve resilience and the overall culture during this time. The choice was to bring forth a positive energy and a sense of personal control through mindfulness and well-being practices, which would benefit students, campus, and the region.

### Starting Small: Campus Momentum, Including Forming a Working Group

That fall (2015) a working group was formed consisting of the campus champions, that is, those who had attended the GGSC’s Summer Institute at Berkeley. This working group held information sessions open to the campus community which were very well attended. Participants shared what they had learned and what they hoped to implement and change, invited others to become involved, and promoted the application process for new individuals to attend the GGSC Summer Institute for Educators in 2016. It is important to note that this initial work was implemented with no designated university funding; these champions volunteered their time to share their experiences and build interest and momentum among others.

During the first academic year (2015–16) the working group was committed to implementing learning in each of the following areas:

1. *Expanding the Circle:* The working group began meeting twice monthly, presenting possibilities for UW-Superior mindfulness and well-being practices with the focus on faculty and staff. In addition, applications were evaluated for potential participants to attend the 2016 GGSC Summer Institute (again, supported financially by the previously mentioned UW-Superior alumna and her husband).
2. *Building Campus Capacity:* Members of the working group presented multiple workshops for the Center for Excellence in Teaching and Learning (CETL). In addition, innovation initiatives focused on embedding mindfulness and well-being into curriculum, beginning with the Teacher Education and Social Work academic programs. Outside of the classroom, workshops and information sessions were being implemented into the university’s Student Affairs and Athletics programs.
3. *Broadening Campus Outreach:* Adding five new members to the working group allowed expansion into additional curriculum areas. The expanded working group developed a Community of Practice, which is defined as a group of passionate individuals who interact regularly while sharing a concern for what they do while learning to do it better (Lave & Wenger, 1991). They offered monthly discussion groups on topics related to well-being, and expanded partnerships with additional academic and non-academic departments. Discussions were also begun with the library to develop a section of resource materials on topics related to well-being.

### Exploring and Securing Funding

Prior to exploring funding options, the working group continued to meet regularly, adding new members returning from the 2016 Summer Institute. All members held full-time positions at the university, and it was through sheer commitment and passion that they contributed to this effort. Everything done was on a grassroots level.

1. In 2017, the working group began to investigate sources of funding to expand its reach and to hire a part-time coordinator, who would do research, carry out administrative duties, and provide service offerings.
2. In that same year, the university initially committed to providing 50 percent release time for one member of the working group to serve in the coordinator position. Once the coordinator position was created, the university established a small working budget for programming.
3. In 2018, hiring the part-time coordinator created an increase in momentum, allowing the university administration to actively seek outside funding.
4. Also, in 2018, through its sustained efforts on campus, the working group received a gift from the same gracious alumna for program services. Simultaneously, the university increased their financial commitment for a full-time center director and physical space. It was at this point the vision of creating a center moved toward actualization. With the hiring of the interim center director, the working group became the internal advisory board.
5. In 2019, the university hired a full-time program manager for the initiative.

### Mission, Vision, Values, and Engagement of the Pruitt Center for Mindfulness and Well-Being

The Pruitt Center for Mindfulness and Well-Being at the University of Wisconsin-Superior was officially established on August 27, 2018. Its mission is *to promote and enhance the science and practice of mindfulness and well-being for students, faculty, staff, and surrounding communities*. In higher education, students must be considered the highest priority. However, research indicates that faculty and staff well-being have a direct correlation to students’ well-being (Cox et al., 2018; Harding et al., 2019; Lovewell, 2012; Hanh & Weare, 2017).

As the foundation of the services and programs offered, one of the earliest members of the working group developed the “PERMANENT” Model of Well-Being (Pruitt Center for Mindfulness & Well-Being, n.d.), which maintains similar domains from one existing science- and practice-based well-being model (PERMA, Seligman, 2011) while also implementing several new ones. The PERMANENT Model of Well-Being incorporates the following nine domains to create a holistic approach: **P**resent Moment Awareness, **E**motional Intelligence, **R**elationships, **M**eaning, **A**chievement, **N**eeded Sleep, **E**xercise, **N**utrition, and **T**hinking, which will be discussed in more detail in a future section.

In creating the vision, the question asked was, “Where do we want to be in ten years?” This question was answered by establishing the vision statement “to become nationally recognized for the integration and advancement of mindfulness and well-being in higher education.” To be comprehensive with the distribution of this material and good community partners, it was determined that the Pruitt Center would also provide opportunities for the greater community to participate and learn.

The Pruitt Center believes that regardless of discipline or position, everyone on campus can be teachers and learners of well-being. This commitment to well-being encourages all members of our campus and local community to learn what it means to be mindful, to improve relationships, to manage stress and adversity, to develop a range of interests and strengths, and to establish a sense of meaning and purpose in each person’s life and career.

The values state that, at its best, education has a dual purpose: building intellectual growth and fostering the development of well-being. This dual purpose provides individuals with the knowledge and skills for career preparation along with the abilities to live authentically with greater meaning and purpose.

The Pruitt Center for Mindfulness and Well-Being also exists as a gathering hub, providing mindfulness and well-being resources and expertise, learning projects, and personal/professional development activities. The Pruitt Center is committed to advancing the science and practice of mindfulness and well-being, utilizing a multidimensional framework that recognizes a diverse population within the UW-Superior campus and surrounding region. The Pruitt Center also focuses efforts on establishing partnerships and collaborations across campus and the greater community.

### Pruitt Center Services, Programs, and Impact

Table 1 summarizes the steps taken in the creation of the Pruitt Center. This section explains the steps taken and the goals of such programming in greater detail.

**Table 1:** UW-Superior's Action Steps to Create the Pruitt Center for Mindfulness and Well-Being

1. Identify Champions and Increase Knowledge
• Identify faculty and staff with a passion and desire to promote and enhance mindfulness and well-being practices
• Invest in advancing the science-based knowledge and practice
2. Form a Working Group
• Champions volunteer time and expertise
• Implement initial work; can be done with *no designated funding*
• Meet on a consistent basis to discuss and plan programming
3. Build Campus Momentum
• Hold information sessions sharing knowledge and practices
• Implement and partner with entrusted, interested departments and programs
• Monthly discussion groups on well-being
• Library resource materials related to well-being
• Expand the circle: additional campus members’ involvement
4. Secure Funding – Foundation and/or through university commitment
• Fundraising, campaign, or other funding opportunities (programming)
• Institutional financial commitment (salaries)
• Explore local and national grant opportunities
5. Create Mission, Vision, Values, and Engagement Statements
• Direction: work being done day to day
• Deliberate wording: *science* and *practice*
• Inclusive and diverse
• Identify or create a holistic model of well-being to guide services and programming
6. Creation of Center
• Atmosphere – warm, inviting, compassionate, and loving
• Central location – visible and accessible to all
• Knowledge resource – campus and greater community
7. Pruitt Center Services, Programs, and Impact
• Students, faculty/staff, greater community
• Science-based
• Practice
• Thriving and flourishing

The bi-annual gratitude visits contribute to strengthening campus relationships and improving morale. The Pruitt Center staff, along with the university Chancellor and Advisory Board, personally distribute hot beverages to faculty and staff in different departments on campus, thanking them for the work they have performed during the semester.

Mindful Mondays, along with yoga and tai chi sessions, have been opportunities for the entire campus community to implement the practice of present moment awareness. In addition to these bi-weekly meetings, each semester Koru Mindfulness (Greeson et al., 2014) and Mindfulness-Based Stress Reduction (MBSR) (Kabat-Zinn & University of Massachusetts Medical Center/Worcester, 1990) are offered, both of which are evidence-based programs. Koru is a four-week mindfulness course designed specifically for emerging adults while MBSR is an eight-week course that is offered to students, faculty, staff, and the greater community. Evaluations of these two mindfulness programs have resulted in high participant satisfaction and recommendations for future Pruitt Center mindfulness programs and events.

Every mindfulness practice is usually offered in-person but have been provided virtually since spring of 2020 because of temporary restrictions due to the Covid-19 pandemic. During the fall of 2020, a virtual fall speaker series was created, which was well-received and had a broader geographic reach. Through those evaluations and post-participant surveys, 100 percent of respondents indicated they would attend a future Pruitt Center event. Another positive effect of this change to an online format has been an expanded geographic reach due to the virtual format, with no limitations to the local area. In addition, yoga and MBSR programs offered elsewhere are often priced too high for community members who would most benefit from them. A primary objective of the Pruitt Center has been to provide services to all people, regardless of socio-economic status.

As part of an ongoing collaboration between the Pruitt Center and the UW-Superior’s Jim Dan Hill Library, a thoughtfully curated collection of mindfulness and well-being books, journals, magazines, and DVDs was created (Jim Dan Hill Library, n.d.). This collection is used for both curriculum and student and staff research. In addition, the Pruitt Center was deliberate in creating its own resource center collection, one that was specifically based on the PERMANENT Model of Well-Being.

Much of the Pruitt Center’s momentum and ability to achieve a presence has been due to the partnerships and collaborations established, both on and off campus. On campus, this reach has included both academic and non-academic departments. Partnerships with over fifteen academic departments include Teacher Education, Social Work, Legal Studies, Psychology, and Health and Human Performance, and over ten non-academic departments, such as Student Affairs, Equity, Diversity, and Inclusion (EDI), Student Success, Residential Life, and Athletics. These collaborations utilize a continuum of services provided by the Pruitt Center staff, including trainings, workshops, mentoring faculty, and implementing mindfulness and well-being into curriculum.

The Pruitt Center was also instrumental in creating a culture of mindfulness and well-being in one of UW-Superior’s Residential Living Communities. Activities offered include: mindfulness, yoga, smoothies for stress-reduction, healthy cookbooks, healthy cooking demonstrations, and movie-watching nights followed by discussions.

Off-campus, the Pruitt Center staff have become a well-respected resource to the greater community. Invitations to give keynote talks and workshops have been extended from local chambers of commerce, human service and non-profit organizations, as well as regional two- and four-year colleges and universities. The variety of workshops and trainings offered surround the nine domains of the PERMANENT model, collectively or separately. The staff have also worked extensively with the local K-12 school districts, offering both in-service professional development trainings and programming for family nights. The Pruitt Center has established an educational cohort with teachers interested in practicing and incorporating mindfulness activities both in and out of the classroom. In the spring of 2020, the Pruitt Center was recruited by the local county treatment courts to provide virtual mindfulness practices as a response to the mandatory shutdown by the governor due to Covid-19. This shutdown significantly limited the access participants had to the valuable weekly support systems offered by the courts. These virtual mindfulness interventions were one way to provide support for this vulnerable population.

### Levelhead-Ed

One partnership to highlight is with Levelhead-Ed, a functional mindfulness app company (Levelheaded, n.d.). This app has a *Learning to Thrive* program with short (3–5 min) daily exercises to which students listen and learn about habits of positive psychology. Students may then begin incorporating these skills into their lives from categories such as stress and anxiety, gratitude, focus and attention, and self-compassion.

More than 350 UW-Superior students have been involved in this effort since Fall of 2019, including the largest cohort Levelhead-Ed has launched to date on a single campus in one semester, Spring of 2020. It was also the first time the company had ever included two entire athletic teams anywhere in the country. During that semester, in addition to the two athletic teams, the Pruitt Center also incorporated Levelhead-Ed into nine classes and one intramural student worker group. A tenth academic class was added with the move to distance learning in mid-March due to Covid-19.

At the end of the semester, 96 percent of all participating students had completed a total of 21,641 exercises on the app, with an average of 74 exercises per student. According to Saundra Schrock, the co-founder of Levelhead and author of the whitepaper *Three Case Studies: Integrating Mindfulness-based Micro-Lessons into Higher Education* (2020), “Based on the findings from the survey, it appears that the students believe that they acquired skills to help them manage stress and anxiety, which is especially important as we navigate the Covid-19 crisis” (p. 54). In addition, 60 percent or greater of the students surveyed responded that their perceived focus and attention had been increased with tools provided by the program.

## The Pruitt Center’s PERMANENT Model of Well-Being

The following section will focus on the creation of the PERMANENT Model of Well-Being, a process which began in 2016. In developing the model, a deliberate effort was made to address the common concerns UW-Superior and many other higher education institutions were facing at that time and continue to face: achievement gaps, student retention, increases in mental health concerns, and career preparation. Learning well-being skills is crucial to an academic education and one way to enhance learning is by encouraging students and staff to perform at their best: physically, emotionally, and mentally (Seldon & Martin, 2017). Lucas and Rodgers (2016) stated, “the purpose of a college degree is to prepare students to be engaged and responsible citizens and to equip them with knowledge and skills to live their lives authentically with greater meaning and purpose” (p.191).

A primary goal of the Pruitt Center was to develop a well-being model founded on current evidence-based practices of mindfulness and well-being. This model would serve as a critical tool in planning, implementing, and evaluating the impact of embedding mindfulness and well-being across all levels of the university and the greater community. The model was designed to be distinct from the traditional, psychological approach to problems, which tend to be “downstream,” or reactive and responsive after problems or illness have already arisen. The “upstream” approach, which is proactive and preventative, aims to encourage well-being while also preventing illness from occurring in the first place (Heath, 2020). This model was designed to enhance the whole person in mind, body, and spirit, expand the pursuit of higher education to also include student well-being; and enable students, faculty, and staff to thrive. “Thriving is defined as being fully engaged intellectually, socially, and emotionally in the college experience” (Schreiner, 2010, p. 2). The model would be simple to understand, diverse, inclusive, and easily implemented in multiple settings. Each domain has well-established evidence improving mental health and well-being by leading researchers in the field. However, because it is a new model, it has not been empirically tested as a theory by the Pruitt Center itself, nor has it been described in any other journal article to date.

Promoting and enhancing students’ well-being during college can lead to a greater likelihood of degree completion and positive effect on their emotional and psychological health (Buck et al., 2008; Oades et al., 2011). One of the most compelling reasons for universities and colleges to make commitments to well-being is the growing concerns surrounding college mental health. According to the *National College Health Assessment Study* (American College Health Association, 2019), college students reported that in the last 12 months: 65.7 percent experienced overwhelming anxiety, 45.1 percent felt so depressed it was difficult to function, and 13.3 percent seriously considered suicide. Additionally, there is evidence that Covid-19 has impacted younger adults disproportionately. Statistics released by the Centers for Disease Control and Prevention (Czeisler et al., 2020) reported that survey respondents aged 18–24 were among the highest subgroups of the 10.7 percent who had seriously considered suicide during the month of June 2020. Because most universities revolve around a high-striving culture, it is easy for students, faculty, and staff to neglect social relationships, emphasize grades and graduation rates, work excessive hours, and engage in other patterns of behavior that diminish well-being (Oades et al., 2011).

In 2016, the lead author began investigating different well-being models: PERMA (Seligman, 2011); Tecmilenio Well-being in Happiness Ecosystem, referred to as “Ecosystem” (Escamilla, 2017); Psychological Well-Being (Ryff & Keyes, 1995); Geelong Grammar School Positive Education (Norrish, 2015); GREAT DREAM (King, 2017); and Subjective Well-Being (Diener, 1984). This led to concerns regarding limiting factors for application in higher education in each of these existing models, except for Tecmilenio’s Well-being Ecosystem (Escamilla, 2017). Much of the research surrounding implementation of well-being models was specific to K-12 education, or represented more white-collar, privileged institutions of higher education, excluding those who serve high populations of first generation and Pell Grant recipients, which is a significant percentage of the student body UW-Superior serves.

### Envisioning the PERMANENT Model of Well-Being

Of all the models explored, Martin Seligman’s PERMA Model (2011) had been researched and implemented most often in educational settings. However, the models researched, including the PERMA Model (Seligman, 2011) and the Ecosystem (Escamilla, 2017), seemed incomplete, lacking certain vital components pertinent to the ability to flourish in higher education. There are four major differences the authors will be discussing as to why the decision was made to create a modified, more holistic model with several new components of well-being specific to the Pruitt Center. The components important to cover include 1) each domain of the model (specifically the similarities and differences to the PERMA Model and the Ecosystem), 2) the name and meaning behind the acronym of the model, 3) integrating the greater community in the PERMANENT Model of Well-Being, and 4) the process included as part of the model.

1. A Description of Each Domain of the Three Models

For comparison’s sake, the authors will be discussing the PERMA model (Seligman, 2011), Tecmilenio’s Well-being in Happiness Ecosystem (Escamilla, 2017), and the PERMANENT Model of Well-Being. As shown in Table 2, some of the domains remained the same or similar, some domains were expanded upon or were changed, while still other critical domains were added. Research related specifically to higher education will be included to validate choices made to change or add to these domains.

**Table 2:** PERMA, Well-being in Happiness Ecosystem, and PERMANENT Model of Well-Being

PERMA Seligman (2011)	Well-being in Happiness Ecosystem Escamilla (2017)	PERMANENT Barker (in development since 2016)	
**P**ositive Emotions	**P**ositive Emotions		**P**resent Moment Awareness
**E**ngagement	**E**ngagement		**E**motional Intelligence
**R**elationships	**R**elationships		**R**elationships
**M**eaning	**M**eaning		**M**eaning
**A**ccomplishment	**A**ccomplishment		**A**chievement
	Physical Well-Being		**N**eeded Sleep
	Mindfulness		**E**xercise
	Character Strengths		**N**utrition
			**T**hinking

Table 2 gives a clear comparison between the three models: PERMA Model (Seligman, 2011), Tecmilenio’s Well-being Ecosystem (Escamilla, 2017), and Barker’s PERMANENT Model of Well-Being. The short comparisons described in the paper will clarify the similarities, differences, and additions, including the rationale of why Barker created and expanded the PERMANENT Model

- **P:** In both the PERMA Model (Seligman, 2011) and the Ecosystem (Escamilla, 2017), the “P” stands for **Positive Emotions**. Within the PERMANENT Well-Being Model, the “P” stands for **Present Moment Awareness**, otherwise known as mindfulness, which is the foundation of this model. Present Moment Awareness is foundational due to the fact it 1) is its own domain and 2) has beneficial impacts on each of the other domains in the model. While mindfulness is incorporated in the Ecosystem, it is not the foundation.

Present Moment Awareness has a strong influence on being, feeling, and functioning well in mind, body, and spirit. At the most fundamental level, mindfulness is based on awareness and attention, which are the prerequisites for understanding or doing anything, or at least for doing anything well, including well-being (Hassed & Chambers, 2015). In the article *Toward the Integration of Meditation into Higher Education: A Review of the Research*, researchers Shapiro et al. (2008) state that mindfulness is noted as contributing to enhanced cognitive and academic performance (including attention and concentration), management of academic stress, and the development of the “whole person.” David and Sheth (2009) discuss many benefits of teaching mindfulness in education: it supports readiness to learn, strengthens attention and concentration, provides tools to reduce stress, reduces anxiety before testing, promotes self-reflection and self-calming, improves impulse control, fosters pro-social behavior and healthy relationships, and supports holistic well-being. Hawkins (2017) states, “learning how to deepen our attention and become more self-aware can enhance our academic capacities and make learning more relevant and impactful” (p. 3).

- **E:** In both Seligman’s (2011) PERMA Model and Escamilla’s Ecosystem (2017), the “E” stands for **Engagement**. While engagement includes mindfulness, the authors believe that Present Moment Awareness allows a person to be engaged, and therefore included engagement in the first domain of the PERMANENT Model. Because of this, the “E” in the PERMANENT Model stands for **Emotional Intelligence**. Whether or not academic content is identified, processed, and remembered is determined by emotions. Emotions also support students finding meaning and purpose and support increasing persistence (Brackett, 2019). According to the book, *The Student EQ Edge*, emotional intelligence can be taught and increased, resulting in improved student success, graduation rates, and career placement and advancement (Stein et al., 2013). One of the major differences between the first two models and the PERMANENT Model is that while positive emotions are essential to learning and well-being (Fredrickson, 2013), knowing how to recognize and regulate difficult emotions also matters.

- **R:** The “R” is for **Relationships** and is the same in all three models (Seligman, 2011; Escamilla, 2017; and Barker’s PERMANENT).

Diener and Seligman wrote in the article *Very Happy People* (2002), *“The most salient characteristics shared by the 10 percent of individuals with the highest levels of happiness and the fewest signs of depression were their strong ties to friends and family and commitment to spending time with them.*” Human beings are naturally social and are literally wired to connect. A meta-analysis of over 100 studies found that teachers who had high quality relationships with their students had 31 percent fewer behavioral problems over the course of the school year than a teacher who did not (Marzano et al., 2003).

- **M:** The “M” is for **Meaning** and is the same in all three models (Seligman, 2011; Escamilla, 2017; and Barker’s PERMANENT).

Meaning is found in both connectivity and contributing to something greater than oneself, such as values, work, family, volunteering, religion, and nature. “Success lies in leveraging one’s unique gifts and talents and becoming the best versions of one’s self” (Schreiner et al., 2012). Individuals experience meaning by developing and using their signature strengths. The VIA Classification of Character Strengths is the language in which character strengths are identified, discussed, and developed (Peterson & Seligman, 2004). “Character strengths not only act as broad protective factors, preventing or mitigating psychopathology and problems, but also enable conditions that promote thriving and flourishing.” (Park, 2015).

- **A:** The “A” is for **Accomplishment** in both PERMA (Seligman, 2011) and the Ecosystem (Escamilla, 2017). In Barker’s PERMANENT Model, “A” stands for **Achievement**. While similar, the main difference is that the earlier two models focus mainly on goals, while the PERMANENT Model of Well-Being also includes failure as part of this domain. As Dr. Tal Ben-Shahar (2011) writes in *Being Happy*, “Failure is essential in achieving success—though it is of course not sufficient for achieving success. In other words, while failure does not guarantee success, the absence of failure will almost always guarantee the absence of success. Those who understand that failure is inextricably linked with achievement are the ones who learn, grow, and ultimately do well. *Learn to fail, or fail to learn*” (p. 29). Failure is an important part of the learning process and should be included as such.

The last four domains in the PERMANENT Model of Well-Being are not included in the PERMA Model (Seligman, 2011), however, the three physical domains are recognized in the Ecosystem (Escamilla, 2017). The final cognitive domain (Thinking) is unique to the PERMANENT Model of Well-Being. The authors believe the added four domains in combination create a more holistic model. Included in each of these domains is research specific to validate the PERMANENT Model of Well-Being in higher education.

- **N:** The “N” in the PERMANENT Model of Well-Being is for **Needed Sleep**. Sleep improves a student’s ability to learn, memorize, and make logical decisions (Killgore et al., 2008). Sleep deprivation decreases a person’s emotional intelligence, assertiveness, self-regard, empathy, and sense of independence, while also affecting the quality of positive thinking, interpersonal relationships, and impulse control. Sleep disturbance further contributes to all major psychiatric conditions, including depression, anxiety, and suicidality (Walker, 2018). Each additional day per week on average a student who experiences sleep problems raises their probability of dropping a course by 10 percent and lowers GPA by 0.02 (Hartmann & Prichard, 2018).

- **E:** The “E” in the PERMANENT Model of Well-Being is for **Exercise**. Exercise has been shown to enhance cognition, aid in detoxification, increase energy, reduce stress, regulate metabolism, fight anxiety and depression, enhance mood and sleep, and improve learning, retention rates, and GPA in college students (Chatterjee & Bell, 2018; Gervais & Caroll, 2020; Rath, 2013; Williams, 2019). Ratey (2013) writes in *Spark: The Revolutionary New Science of Exercise and the Brain* that exercise enhances learning on a variety of levels by improving alertness, attention, and motivation. It also prepares and encourages nerve cells to fasten to one another, while helping the brain prepare to learn and retain more information.

- **N:** The “N” in the PERMANENT Model of Well-Being is for **Nutrition**. Poor eating habits adversely impact academic performance, while healthy dietary behaviors are favorable predictors of academic success. For college students who consume a larger amount of fruits and vegetables, GPAs were enhanced by as much as 0.15. It has also been proven that consuming breakfast strengthens scholastic achievement and is fundamental in developing a wholesome positive lifestyle (Burrows et al., 2017).

- **T:** The “T” in the PERMANENT Model of Well-Being is for **Thinking**. Cognitive Behavioral Therapy (CBT) founders Aaron Beck and Albert Ellis created concepts, techniques and teachable/learnable skills initially developed for psychotherapy (Beck, 1967; Ellis, 1997). These concepts are now being taught and used proactively, helping to build resilience, prevent psychological disorders, and improve and enhance well-being. The PENN Resiliency Program, which is based on CBT principles, is effective in reducing and preventing anxiety and depressive symptoms, hopelessness, and conduct problems. It has also been used scientifically to increase optimism, effective problem solving, sense of meaning, self-efficacy, flexibility, impulse control, empathy and close relationships (Masten & Reed, 2002; Reivich & Shatté, 2003; Gillham et al., 1995; Gillham et al., 2006).

2. The Name and the Meaning behind the Acronym

Deep thought went into the naming of the PERMANENT Model of Well-Being, not only regarding the domains included but the naming of the model itself. Unlike the other models, the added benefit of the acronym is the word itself: PERMANENT. Permanent is defined as “continuing or enduring without fundamental or marked change. Not easily removed, washed away, or erased. Long-lasting” (Merriam-Webster, n.d., Definition 1-2a). One of the goals of establishing this well-being model is to create permanent, long-lasting, positive well-being habits through the means of neuroplasticity. According to Schwartz and Gladding (2011), “neuroplasticity includes any process that results in a change in the brain’s structure, circuits, chemical composition, or function in response to changes in the brain’s environment” (p. 36).

3. The Inclusion of the Greater Community

One component of the University of Wisconsin-Superior’s Mission Statement, as well as the Pruitt Center’s own Mission Statement, is to engage the community and surrounding region. Many of the models researched focused on populations specific to educational institutions (students, faculty, and staff), while the Pruitt Center made a deliberate effort to serve all populations: students, faculty and staff, *and* the greater community. The information included in the PERMANENT Model of Well-Being was too important not to share with as many individuals as possible, including the greater community.

4. The PERMANENT Process: Learn, Experience, Reflect, Repeat

There are four important scaffolding levels for the PERMANENT Model of Well-Being to be fully realized and beneficial: *learn, experience, reflect,* and *repeat*. The first level is providing and enhancing an individual’s knowledge with empirical research on the benefits and limitations of well-being. Many individuals still believe that well-being is synonymous with self-help and not backed by research, and therefore need more information before feeling comfortable implementing the suggested strategies into their own personal and professional lives.

Well-being is unique in that a person needs to learn how to be well, and to practice well-being (Morris, 2009). Individuals must experience well-being to feel first-hand the benefits of taking action, doing things differently, and possibly feeling uncomfortable in the process.

Reflection allows a person to go deeper within themselves and the experience itself. It allows the permission and space to ask important questions such as, “How well did that work? What did you notice about yourself and others? How will this negatively or positively affect the mind, body, and spirt?” Through both writing and engaging in meaningful conversations where individuals feel safe allows them to gain insights and feedback.

Well-being requires hard work of both decreasing bad or unhealthy habits, along with creating and enhancing new positive habits. One way of doing this is through what Schwartz and Gladding (2011) call “self-directed neuroplasticity.” This consists of focused attention, hard work, commitment, and dedication to direct choices that rewire a person’s brain to work for them.

There is nothing magical about the time required for habit formation; it is the frequency that makes the difference. It is better to do a little of something every day—the daily practice—whether it be training for a marathon, studying for an exam, or learning how to meditate. This is the only way to learn any new skill. This is the practice. Table 3 shows the domains of the PERMANENT Model of Well-Being, the programming and activities offered by the Pruitt Center, the learning/insights, and the leaders in each of the fields.

**Table 3:** Programs Offered with the PERMANENT Model of Well-Being as the Basis

PERMANENT Model of Well-Being	Programming/Activities Offered	Learning/Insights	Leaders in the Field
Present Moment Awareness (Mindfulness)	• MBSR • Koru • Mindfulness offerings ○ Mindful Monday ○ Yoga Classes ○ Tai Chi Classes • Workshops • Speakers • Levelhead-Ed • Movie screenings (*Mindfulness Movement*) • Book club (*Mindful of Race*)	• Self-awareness • Increased focus and attention • Stress reduction • Enhanced cognitive and academic performance • Self-compassion	• Jon Kabat-Zinn (MBSR) • Holly Rogers (Koru) • Shauna Shapiro • Richie Davidson • Kristin Neff • Ellen Langer
Emotional Intelligence	• Emotional Intelligence course • EQi2.0 Assessment • Workshops • Speakers • Movie Screening (*Inside Out*) • Book Club (*Empathy*) • Elementary school visits with book *Ways to Be from A to Z*	• Emotional literacy • Emotional regulation • Emotional contagion – both positive and negative • Savoring • Gratitude • Awe • Positivity	• Marc Brackett • Barbara Fredrickson • Daniel Goleman • Peter Solvay • John D. Mayer
Relationships	• Residential Living Community • Open Communication Forums ○ Understanding Covid-19 • Campus Gratitude Days • Workshops • Speakers • Book Clubs (*Mindful of Race*, *Dare to Lead*)	• Active Listening • Active/Constructive Responding • Kindness • Establish social support • Building of community	• John Gottman • Ed Diener • Martin Seligman • Christopher Peterson
Meaning	• Workshops • Post-Traumatic Growth • Speakers • Volunteerism • Service Learning • Movie Screenings (*Happy* and *I Am*)	• Values • Job, Career, Calling • Serving goals greater than oneself • Altruism	• Viktor Frankl • Amy Wrzesniewski • Parker Palmer • William Damon
Achievement	• Workshops ○ VIA Character Assessment ○ Signature Strengths ○ SMART Goals ○ Grit ○ Growth and Fixed Mindset • Speakers	• Identify goals • Cultivate strengths • Reframe failure to learning • Hope theory • Grit • Goals • Growth mindset • Failure	• Tal Ben-Shahar • Ryan Niemiec • Angela Duckworth • Carol Dweck • Rick Snyder
Needed Sleep	• Workshops ○ Sleep hygiene ○ Establishing sleep routines ○ Stress and sleep ○ Sleep and performance • Speakers • Movie Screening (*Fully Charged*)	• Recovery • Mood – Physical/Cognitive enhancement • Establish sleep routine • Sleep Hygiene • Peak performance • Individualize – find your fit	• Roxanne Prichard • Matthew Walker • Arianna Huffington • Tom Rath
Exercise	• Yoga Classes • Tai Chi Classes • Walking group • Workshops • Speakers • Movie Screening (*Fully Charged*)	• Mood – Physical/Cognitive enhancement • Play – reframe as fun • Nature • Peak performance • Individualize – find your fit	• John Ratey • Tom Rath • Michael Gervais • Rangan Chatterjee
Nutrition	• Workshops • Speakers • Healthy cooking demonstrations • Healthy recipes cookbook • Movie Screening (*Fully Charged*)	• Mood – Physical/Cognitive enhancement • Mindful eating • Food origin/sustainability • Peak performance • Individualize – find your fit	• Rangan Chatterjee • Tom Rath • Michael Pollan • Leslie Korn
Thinking	• Workshops: tenets of positive psychology ○ Resilience ○ Optimism ○ CBT ○ Choice ○ Best self • Speakers • ABCD Model • Elementary school visits with book *Ways to Be from A to Z*	• Reframing • Gratitude • Self-talk • Metacognition • Choice	• Martin Seligman • Karen Reivich • Albert Ellis • Aaron Beck • Rick Hanson • Jeffrey Schwartz

### Obstacles and Challenges in Creating the Pruitt Center for Mindfulness and Well-Being

As with any new endeavor, there are always obstacles and challenges to overcome. Included in this section are a few the Pruitt Center encountered, the exploration of which could be beneficial to others interested in creating a center of well-being on their campus.

Acquiring upper administration support is critical in pursuit of creating a center. Providing research on how mindfulness and well-being can contribute to reducing mental health concerns, improving academic performance, increasing retention and recruitment is helpful in attaining the support needed.

Integrating mindfulness and well-being into curriculum is also beneficial regardless of subject area. Not all faculty are open to incorporating unfamiliar areas into their curriculum, especially topics of which they themselves might not feel comfortable. Creating opportunities for these faculty members to learn and practice is one way to overcome this resistance.

Empirical data is important and often overlooked in measuring change and effectiveness where mindfulness and well-being are concerned. Establishing a baseline and assessing effectiveness despite the newness of the center and the small size of the staff was a major challenge. The Pruitt Center did what it could with the resources it had, including implementing pre- and post-surveys, satisfaction surveys after special events, and sending out the annual Wake Forest Well-Being Assessment (Wake Forest Wellbeing Collaborative, n.d.) to our traditional undergraduate population. The plan is to incorporate more evaluation and research as the center grows.

The Pruitt Center made a conscious decision to serve three different populations—students, faculty/staff, and greater community—all with unique needs and wants. This choice may not be suitable for every institute of higher education. It would be appropriate to thoughtfully identify populations served.

### The Future of the Pruitt Center of Mindfulness and Well-Being

The University of Wisconsin-Superior began the process of creating and implementing a three-year strategic plan in 2020, with the intention of implementing the plan in 2021. The Pruitt Center is proud to be included in this plan to support the well-being of our campus community. There are two objectives of the strategic plan to which the Pruitt Center is central; both are focused on the well-being and thriving of our students, faculty, and staff.

In 2018, the University made the decision to place Student Health and Counseling Services (SHCS) and the Pruitt Center for Mindfulness and Well-Being under one umbrella. This change was implemented in the spring of 2019, overseen by the newly created Director for Health, Counseling, and Well-Being. The decision to place both SHCS and the Pruitt Center under the same leadership was based in part on Corey Keyes’ *Mental Health Continuum Model*, which views mental health and mental illness on one axis with flourishing and languishing on the other (2002). Too often campuses provide only reactive/responsive services, which is therapy and psychiatric services that SHCS provides. However, with the addition of the Pruitt Center, the University was able to extend our services of mental health as a more comprehensive approach and provide proactive/preventative services. The Pruitt Center leadership would eventually like to see mindfulness and well-being become the basis for UW-Superior to be considered a Well-Being University.

## Conclusion

It takes great effort and commitment, both individually and institutionally, to move forward in the area of implementing mindfulness and well-being in higher education. From its humble grassroots beginning, the Pruitt Center has benefitted from the commitment of a dedicated working group and generous funding from an alumna and the university, which, combined, allowed UW-Superior to create a center and continue to fulfill its mission.

There have been, and will continue to be, many challenges facing every institute of higher education. UW-Superior is not immune to these challenges, even with the creation of the Pruitt Center and our commitment to mindfulness and well-being. These obstacles affecting all of higher education include: declining enrollment, budget cuts, program cuts, increased work-loads, and increased mental health concerns, all of which have a significant impact on the well-being of an entire campus community. In 2020, these concerns have been amplified even further given the climate of Covid-19 globally and political and racial unrest nationally. These are exactly the reasons that well-being must be addressed, promoted, and implemented into the campus culture.

We can no longer afford to approach these obstacles in the same reactive way we have in the past and expect different outcomes. Higher education must consider investing in creating a culture which prioritizes the proactive/preventative skills of mindfulness and well-being and valuing the whole-person. A sustained commitment to this approach holds promise to enable students, faculty, staff, and surrounding communities to live a life with meaning and purpose, and to flourish, both personally and professionally.

## Data Availability

Not applicable.
